# Autoinflammation and immunodeficiency

**DOI:** 10.1186/1546-0096-12-S1-I34

**Published:** 2014-09-17

**Authors:** Joost Frenkel

**Affiliations:** 1 Paediatrics, University Medical Center Utrecht, Utrecht, Netherlands

## 

Autoinflammatory diseases are characterized by more or less spontaneous inflammation without inciting infection or autoimmunity.

These can be either acquired or genetically determined. The latter –hereditary– autoinflammatory syndromes have been classified by some as primary immunodeficiencies: defects affecting the control of the innate arm of the immune system. Immunodeficiency syndromes, however, have generally been considered to be defects in host defense, rendering the patient susceptible to infectious diseases.

Even when regarding immunodeficiency and autoinflammation as separate entities, situations do occur where patients suffer both non-specific sterile inflammation and increased susceptibility to infection. This may occur in prototypic autoinflammatory diseases as well as in well recognized primary immunodeficiencies. In addition, there are intermediate disorders that are both autoinflammatory and immunodeficient by nature (table 1).

**Table 1:** 

	Autoinflammatory disease	Mixed disorder	Innate immunodeficiency	Adaptive immunodeficiency
**example**	Mevalonate kinase deficiency	HOIL1-deficiency	Chronic granulomatous disease	Common Variable immunodeficiency Rag1 deficiency
**feature**	Serious bacterial infections	Recurrent fever and humoral immunodeficiency	Sterile granulomata	Sterile granulomata

Diagnosis of infection in autoinflammatory diseases is challenging, as these occur against a background of recurrent fever episodes. Conversely, ruling out infection is a prerequisite for diagnosing autoinflammation in immunodeficiency.

This distinction is relevant for patient management, since some autoinflammatory patients may benefit from antimicrobial prophylaxis, whereas sterile inflammation in immunodeficiency may benefit from approaches such as interleukin-1 blockade.

## Disclosure of interest

J Frenkel Consultant for: Novartis Pharma.

